# PET OWNERSHIP AND MAINTENANCE OF EXECUTIVE FUNCTION IN OLDER ADULTS– EVIDENCE FROM THE BLSA

**DOI:** 10.1093/geroni/igac059.3028

**Published:** 2022-12-20

**Authors:** Erika Friedmann, Nancy Gee, Eleanor Simonsick, Melissa Kitner-Triolo, Erik Barr, Barbara Resnick, Ikmat Adesanya, Merve Gurlu

**Affiliations:** University of Maryland Baltimore, Baltimore, Maryland, United States; Virginia Commonwealth University, Richmond, Virginia, United States; National Institute on Aging, Baltimore, Maryland, United States; Brain Aging & Behavior Section, Baltimore, Maryland, United States; University of Maryland School of Nursing, Baltimore, Maryland, United States; University of Maryland, Baltimore, Maryland, United States; University of Maryland, Baltimore, Maryland, United States; University of Maryland Baltimore, Baltimore, Maryland, United States

## Abstract

Pet ownership (PO) or human-animal interaction (HAI) has been associated with better physical and mental health in individuals with existing disease or disability. In experimental studies, HAI improves some aspects of cognitive function. No research addresses HAI and longitudinal changes in cognitive function in older adults. We examined the relationship of PO to maintaining executive function (EF) within generally healthy community-dwelling older adults in the Baltimore Longitudinal Study of Aging (BLSA). We hypothesized less EF deterioration among pet owners than non-owners; and less deterioration among dog-walkers than owners who don’t walk their dogs. 637 women (55.9%) and men aged 50–100 years (M=68.3, SD=9.6) completed a PO questionnaire which ascertained ownership history and comprehensive examination every 1–4 years over 1–13 years (M=7.5, SD=3.6). Linear mixed models with time varying PO examined changes in EF according to PO. Pet owners (n=185) were younger (p < 0.001) and healthier (p=0.030) than non-owners; thus, age and comorbidities were included as covariates in the longitudinal analyses. EF, assessed using the Trail-Making Test (TMT), deteriorated with age (p’s < 0.001). Deterioration was less severe among pet owners than non-owners (TMT A: p < 0.001; B: p < 0.001; difference: p=0.042) and dog owners than non-owners (TMT A: p=0.025; B: p=0.009; difference: p=0.076), but not among cat owners than non-owners. Among dog owners (n=73), dog walkers experienced less deterioration than non-walkers (TMT A: p=0.156; B: p=0.001; difference: p < 0.001). This study provides the first longitudinal evidence that PO may contribute to maintaining EF among community-dwelling generally healthy older adults as they age.

